# Neurotransmitter Profiles Are Altered in the Gut and Brain of Mice Mono-Associated with *Bifidobacterium dentium*

**DOI:** 10.3390/biom11081091

**Published:** 2021-07-23

**Authors:** Berkley Luck, Thomas D. Horvath, Kristen A. Engevik, Wenly Ruan, Sigmund J. Haidacher, Kathleen M. Hoch, Numan Oezguen, Jennifer K. Spinler, Anthony M. Haag, James Versalovic, Melinda A. Engevik

**Affiliations:** 1 Department of Pathology and Immunology, Baylor College of Medicine, Houston, TX 77030, USA; berkley.luck@gmail.com (B.L.); thomas.horvath2@bcm.edu (T.D.H.); sigmund.haidacher@bcm.edu (S.J.H.); kathleen.hoch@bcm.edu (K.M.H.); numan.oezguen@bcm.edu (N.O.); spinler@bcm.edu (J.K.S.); anthony.haag@bcm.edu (A.M.H.); jamesv@bcm.edu (J.V.); 2 Department of Pathology, Texas Children’s Hospital, Houston, TX 77030, USA; 3 Department of Virology and Microbiology, Baylor College of Medicine, Houston, TX 77030, USA; kristen.engevik@bcm.edu; 4 Department of Pediatrics, Baylor College of Medicine, Houston, TX 77030, USA; wenly.ruan@bcm.edu; 5 Section of Gastroenterology, Hepatology, and Nutrition, Texas Children’s Hospital, Houston, TX 77030, USA; 6 Department of Regenerative Medicine & Cell Biology, Medical University of South Carolina, Charleston, SC 29425, USA

**Keywords:** gut–brain axis, neurotransmitters, LC-MS/MS, gut microbiome, *Bifidobacteria*, GABA

## Abstract

Background: Accumulating evidence indicates that the gut microbiota can synthesize neurotransmitters as well as impact host-derived neurotransmitter levels. In the past, it has been challenging to decipher which microbes influence neurotransmitters due to the complexity of the gut microbiota. Methods: To address whether a single microbe, *Bifidobacterium dentium,* could regulate important neurotransmitters, we examined *Bifidobacteria* genomes and explored neurotransmitter pathways in secreted cell-free supernatant using LC-MS/MS. To determine if *B. dentium* could impact neurotransmitters in vivo, we mono-associated germ-free mice with *B. dentium* ATCC 27678 and examined fecal and brain neurotransmitter concentrations. Results: We found that *B. dentium* possessed the enzymatic machinery to generate γ-aminobutyric acid (GABA) from glutamate, glutamine, and succinate. Consistent with the genome analysis, we found that *B. dentium* secreted GABA in a fully defined microbial media and elevated fecal GABA in *B. dentium* mono-associated mice compared to germ-free controls. We also examined the tyrosine/dopamine pathway and found that *B. dentium* could synthesize tyrosine, but could not generate L-dopa, dopamine, norepinephrine, or epinephrine. In vivo, we found that *B. dentium* mono-associated mice had elevated levels of tyrosine in the feces and brain. Conclusions: These data indicate that *B. dentium* can contribute to in vivo neurotransmitter regulation.

## 1. Introduction

The communication between the intestine and the brain, known as the gut–brain axis, has recently attracted attention for its crucial role in human health. Intestinal microbes are known to modulate both the enteric nervous system (ENS) and central nervous system (CNS) and have been linked to intestinal motility [1,2,3,4,5,6,7,8,9,10], visceral sensitivity [11,12,13,14,15,16,17,18], anxiety [19,20,21,22,23,24], depression [25,26], neurodegenerative disease [27,28], multiple sclerosis [29,30], and autism spectrum disorder [31]. One way gut microbes can interact with the ENS, and CNS is through the modulation of host neurotransmitters and/or related pathways. Consistent with this notion, select microbes have been found to produce a range of major neuroactive compounds including tyrosine, tryptophan, dopamine, norepinephrine, and GABA. Some neuro-active compounds such as GABA cannot cross the blood–brain barrier and have localized effects within the gut. In contrast, other neuro-active molecules such as tyrosine can cross the blood–brain barrier and can affect both the gut and brain. Most of the work focusing on microbial-derived neurotransmitters has been performed in vitro, typically using rich microbial media which may not reflect the conditions of the gastrointestinal tract. Likewise, in vivo studies using gnotobiotic models have largely focused on complex microbial communities that obscure the ability to identify specific microbes driving neurotransmitter changes. As a result, new mono-associated gnotobiotic animal models are needed to dissect how individual microbes influence intestinal and neuronal neurotransmitter levels.

The communication between the gut and brain is speculated to be particularly important during early life. During this early developmental period, the gut microbiota is dominated by the genus *Bifidobacterium* (phylum Actinobacteria) [32,33,34,35,36,37,38]. *Bifidobacteria* species dominant in human infants include *B. longum*, *B. longum subspecies infantis*, *B. breve*, *B. bifidum*, *B. catenulatum*, *B. adolescentis*, *B. angulatum*, *B. animalis*, and *B. dentium* [39,40,41,42,43]. These early-life *Bifidobacteria* have been shown by several groups to beneficially modulate the gut–brain axis. For example, *B. longum* strains NCC3001, 1714, and R0175 can modulate anxiety in animal models [44,45,46], and *B. longum* NCC3001 was found to improve depression scores in patients with IBS [47]. Likewise, *B. infantis* improved depressive-like behaviors in a rat model of maternal separation [48], while *B. longum* 1714 was shown to enhance object recognition and cognition in a mouse model [49]. Our previous work has also demonstrated that neonatal colonization with a consortium of human-derived *Bifidobacterium* species (*B. longum subspecies infantis*, *B. bifidum*, *B. breve*, and *B. dentium*) affects the CNS synaptic plasticity, microglia activation, and rodent behavior, including anxiety and hyperactivity [50,51]. We have also shown that *B. dentium* administration to adult mice modulates the serotonergic system and adult behavior [52]. Although these phenotypic effects have been observed, much of the mechanisms by which *Bifidobacteria* modulate the gut–brain axis remains to be discovered.

To address this knowledge gap, we sought to characterize the neuroactive compounds produced by a single human *Bifidobacteria* species in vitro and in vivo. We selected *B. dentium* because it is found in infants and adults [53], has the metabolic capacity to colonize the intestine [54], possesses anti-inflammatory, epithelial-barrier enhancing properties [55,56], and stimulates serotonin release from enterochromaffin cells [52]. The importance of these functions in human health motivated us to characterize the neurotransmitter profile of *B. dentium*. To effectively address the contribution of *B. dentium* on neurotransmitter concentrations in vivo, we used a gnotobiotic model (defined microbiota applied to germ-free mice); a well-established model for studying the effects of specific microorganisms on the gut–brain axis [20,21,57]. Our data indicates that *B. dentium* colonization is associated with a unique gut and neuronal neurotransmitter profile.

## 2. Materials and Methods

### 2.1. Bacterial Culture

*Bifidobacterium dentium* ATCC 27678 (adult human fecal isolate) was cultured in Man, Rogosa, and Sharpe (MRS; Difco) media in an anaerobic workstation (Anaerobe Systems AS-580) at 37 °C with a gas mixture of 5% CO_2_, 5% H_2_, and 90% N_2_. After culturing in MRS media, *B. dentium* was sub-cultured into a chemically defined media, termed ZMB1 [58], at an optical density (OD_600nm_) of 0.1 and cultured anaerobically at 37 °C for 18 h. Bacterial cultures were centrifuged at 5000× *g* for 5 min and the cell-free supernatant was sterile-filtered (0.2 µm-pore PVDF-membrane, Millipore) for LC-MS/MS analysis.

### 2.2. Mouse Models

Gnotobiotic experiments were approved by the Institutional Animal Care and Use Committee (IACUC) at the Baylor College of Medicine, Houston, TX, USA. The germ-free mice had ad libitum access to irradiated food (Rodent 50 IF/6F auto 5V0F chow cat# 3002875-703, Lab Diet.com) and water. Adult male germ-free Swiss Webster mice (6–9 months of age) were gavaged with sterile MRS (germ-free controls, *n* = 5) or 3.2 × 10^8^ CFU (in 0.2 mL) *B. dentium* ATCC 27678 in MRS (*B. dentium* mono-associated, *n* = 5) and maintained in isolators for 17 days as previously described [52]. For the neurotransmitter and neuroactive compounds studies, only male mice were used to limit variation. Colonization of the mice was confirmed by culturing fecal samples by plating. Feces were collected from mice prior to sacrifice. Mice were euthanized via isofluorane asphyxiation and the whole brain and colon were excised. Tissue was fixed in Carnoy’s fixative and paraffin-embedded.

To confirm bacterial colonization, Gram staining was performed on colon tissue sections. Briefly, tissue sections were dehydrated (xylene, 100%, 95%, 75%, 50% ETOH), incubated with crystal violet, gram iodine, decolorized, and counter-stained with gram safranin. Sections were then incubated with picric acid-acetone. After dehydration through a series of alcohols (75%, 95%, 100% ETOH) and xylene, sections were cover-slipped and imaged on an upright Nikon Eclipse 90i microscope at 20× using a Plan Apo (NA 0.75) differential interference contrast (DIC) objective.

### 2.3. Bifidobacteria Genome Mining

The ability of *Bifidobacteria* members to generate neuro-active metabolites was estimated using the Integrated Microbial Genomes (IMG) database (http://img.jgi.doe.gov (accessed on 17 January 2021)). The IMG annotates publicly available sequence data and the total number of *Bifidobacteria* genomes queried are shown in Supplemental Table S1.

### 2.4. Metabolite Analyses of B. dentium Cultures and Gnotobiotic Mouse Feces and Brain Tissues

#### 2.4.1. LC-MS/MS Equipment

The liquid chromatography-tandem mass spectrometry (LC-MS/MS) system was comprised of a Shimadzu Nexera X2 MP Ultrahigh-Performance Liquid Chromatography (UHPLC) system (Kyoto, Japan) coupled to a Sciex 6500 QTrap hybrid triple-quadrupole/linear ion trap MS system from Danaher (Washington, DC, USA). Operational control of the LC-MS/MS was performed with Analyst^®^ (Ver. 1.6.2), and quantitative analysis was performed using MultiQuant™ (Ver. 3.0.1).

#### 2.4.2. LC-MS/MS Methods

Fecal and tissue homogenization and extraction procedures, and targeted LC-MS/MS-based metabolomics methods used for the quantitative analysis of the glutamate cycle and tyrosine pathway metabolites are described in the Supplemental Section. See Tables S2 and S3 in Supplemental Materials for the molecule-specific selected reaction monitoring (SRM) parameters used to acquire the chromatographic data for the targeted metabolites.

#### 2.4.3. Calibration Standard Preparations

Calibration standards (Calibrators) for the glutamate cycle and tyrosine pathway methods were prepared at concentrations of 0.98, 3.9, 15.6, 62.5, and 1000 ng/mL for all metabolites measured in each method. In each instance, a consistent concentration of method-specific deuterated IS compounds were added to each calibrator and unknown sample—the assay-specific concentration levels for each deuterated IS compound is specified in the Supplemental Materials section.

### 2.5. Statistics

GraphPad Software (GraphPad Software, Inc. La Jolla, CA, USA) was used to generate graphs and analyze data for statistical significance. Comparisons between the groups were made with one-way or two-way Analysis of Variance (ANOVA), using the Holm-Sidak post-hoc test comparisons, * *p* < 0.05.

## 3. Results

### 3.1. Bifidobacteria Harbor the Enzymatic Machinery to Generate the Neurotransmitter GABA

**Figure 1:**
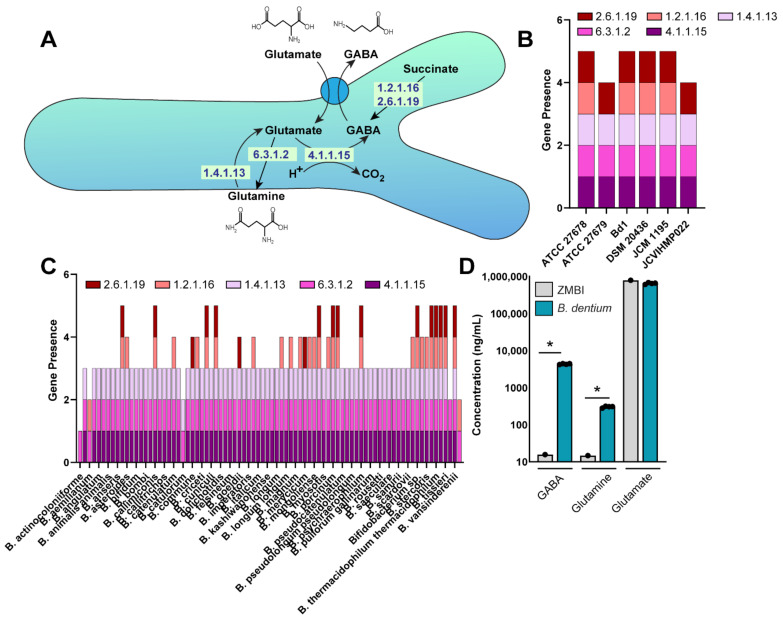
(**A**). Gene pathways in *Bifidobacterium dentium* as identified via KEGG (https://www.genome.jp/kegg/kegg2.html) related to GABA, glutamate, and glutamine production. (**B**). Genome analysis of the JGI Integrated Microbial Genomes (IMG) database (http://img.jgi.doe.gov (accessed on 17 January 2021)) of six *B. dentium* genomes for the enzymes (Enzyme Commission, ECs) involved in glutamate, glutamine, and GABA production. Filled bars represent the presence of at least 1 gene copy of each enzyme. (**C**). Genome analysis of 83 *Bifidobacteria* genomes using the JGI Integrated Microbial Genomes (IMG) database. (**D**). Absolute concentrations of GABA, glutamate, and glutamine in uninoculated ZMB1 and cell-free bacterial conditioned ZMB1 from *B. dentium* after 18 h of growth. *n*= 4 biological replicates, two-way ANOVA, * *p* < 0.05.

*Bifidobacteria* are known to colonize the mammalian gastrointestinal tract, but the characterization of *Bifidobacteria*-specific neuro-active compounds remains incomplete. We first focused on the neurotransmitter GABA, which serves as the major inhibitory neurotransmitter of the ENS and CNS [59]. Select microbes have been demonstrated to generate GABA from glutamate or succinate as a mechanism to decrease intracellular pH [60]. In this process, glutamate is taken into the bacteria via the transporter GadC and can be converted to GABA by the enzyme glutamate decarboxylase (GAD; Enzyme Commission number (EC) 4.1.1.15). Glutamate can also be converted to glutamine (EC 6.3.1.2) or glutamine can be converted to glutamate (EC 1.4.1.13). Alternatively, in some bacteria succinate can be converted into GABA via succinate-semialdehyde dehydrogenase (EC: 1.2.1.16) and 4-aminobutyrate-2-oxoglutarate transaminase (EC 2.6.1.19). To address whether *B. dentium* harbored the genes for the ECs associated with GABA production, we queried the *B. dentium* Bd1 genome in the KEGG database (https://www.genome.jp/kegg (accessed on 17 January 2021)) and found that this genome contained the complete enzymatic pathway to generate GABA from glutamate/glutamine and succinate (Figure 1A). Four of the six *B. dentium* genomes in the Integrated Microbial Genomes (IMG) database (http://img.jgi.doe.gov (accessed on 17 January 2021)), contained all five genes for GABA production from glutamine/glutamate and succinate (Figure 1B). *B. dentium* ATCC 27679 and JCVIHMP022 did not contain the gene to express succinate-semialdehyde dehydrogenase (EC 1.2.1.16) needed to convert succinate to GABA, although these species could still produce GABA from glutamate.

To see how common this pathway was among other *Bifidobacteria* members, we examined the genomes of 83 *Bifidobacteria* species (total 498 genomes) (Figure 1C, Supplemental Table S1). Thirty out of 83 *Bifidobacteria* species contained succinate-semialdehyde dehydrogenase (EC 1.2.1.16) for converting succinate to GABA. Eighteen different *Bifidobacteria* species contained the gene for 4-aminobutyrate-2-oxoglutarate transaminase (EC 2.6.1.19), but not for succinate-semialdehyde dehydrogenase. Seventy-nine *Bifidobacteria* species (94.1%) possessed the genes to generate GABA from glutamate or glutamine (EC 4.1.1.15, 6.3.1.2, 1.4.1.13), suggesting that the majority of *Bifidobacteria* species can only generate GABA from glutamate and not succinate (Figure 1C). Four *Bifidobacteria* species, *B. actinocoloniiforme* (2 genomes), *B. aemilianum* (1 genome), *B. catulorum* (1 genome), and *B. xylocopae* (1 genome) did not have the ability to generate GABA from glutamate (EC 4.1.1.15) or succinate (EC 2.6.1.19), suggesting that GABA production may not be essential for these strains. In this genome comparison, we found that *B. dentium* (6 genomes) and 10 other *Bifidobacteria* species contained all genes required to convert glutamine, glutamate, and succinate into GABA. These findings indicate that *B. dentium* is well equipped to produce GABA.

To address whether *B. dentium* could generate GABA in vitro, we grew *B. dentium* ATCC 27678 in a fully defined bacterial media, termed ZMB1, for 18 h and examined GABA, glutamine, and glutamate levels by LC-MS/MS (Figure 1D). ZMB1 has high concentrations of glutamate (787 µg/mL) and we observed a slight decrease in glutamate concentrations in ZMB1 after the growth of *B. dentium*. While glutamate concentrations were relatively unchanged, we did observe increased concentrations of GABA and glutamine in *B. dentium*-conditioned ZMB1, indicating that *B. dentium* can generate GABA in ZMB1 in vitro.

### 3.2. Bifidobacterium Dentium ATCC 27678 Influences Fecal GABA Levels in Gnotobiotic Animals

**Figure 2:**
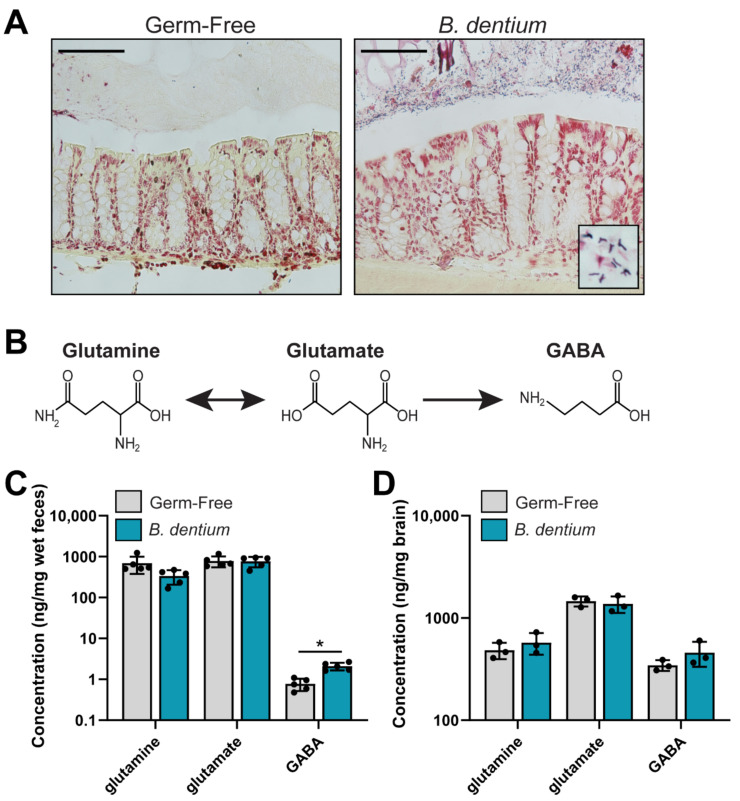
(**A**). Representative Gram stains of the germ-free and *B. dentium* mono-associated colon. Scale bar = 100 µm. (**B**). Depiction of the glutamine, glutamate, GABA pathway. (**C**). Analysis of glutamine, glutamate, and GABA measured from a known mass of stool (ng/mg wet feces weight) by LC-MS/MS reflecting in vivo levels of these neurotransmitters from the germ-free and *B. dentium* mono-associated mice. (**D**). Concentrations of glutamine, glutamate, and GABA in a known mass of whole brain homogenate (ng/mg brain). *n* = 3–5 mice/group; two-way ANOVA, * *p* < 0.05.

After confirming in vitro production of GABA by *B. dentium* ATCC 27678, we mono-associated adult mice to determine if *B. dentium* could modulate GABA in vivo. We examined stool and whole brain homogenates in both *B. dentium* mono-associated mice and germ-free mice. Colonization was confirmed by Gram staining (Figure 2A). As expected, bifid-shaped microbes were found in the mucus layer above the colonic epithelium in the *B. dentium* mono-associated mice, while no microbes were present in the germ-free controls (Figure 2A). Next, we assessed the relative concentrations of fecal GABA, glutamate, and glutamine cycle compounds by LC-MS/MS (Figure 2B,C). We found that *B. dentium* mono-association had no effect on glutamine or glutamate concentrations, both of which were high in the rodent diet (Rodent 50 IF/6F auto 5V0F chow cat# 3002875-703). However, we did observe a significant increase in colonic GABA. We also examined GABA, glutamate, and glutamine in the whole brain homogenate (Figure 2D). Interestingly, no changes were observed in glutamine, glutamate, or GABA concentrations in *B. dentium* mono-associated brains compared to germ-free controls. This is consistent with the notion that GABA is unable to cross the blood–brain barrier. These data indicate that *B. dentium* can impact the concentration of GABA in the colon.

### 3.3. Bifidobacteria Harbor the Enzymatic Machinery to Generate the Neuroactive Compoung Tyrosine

**Figure 3:**
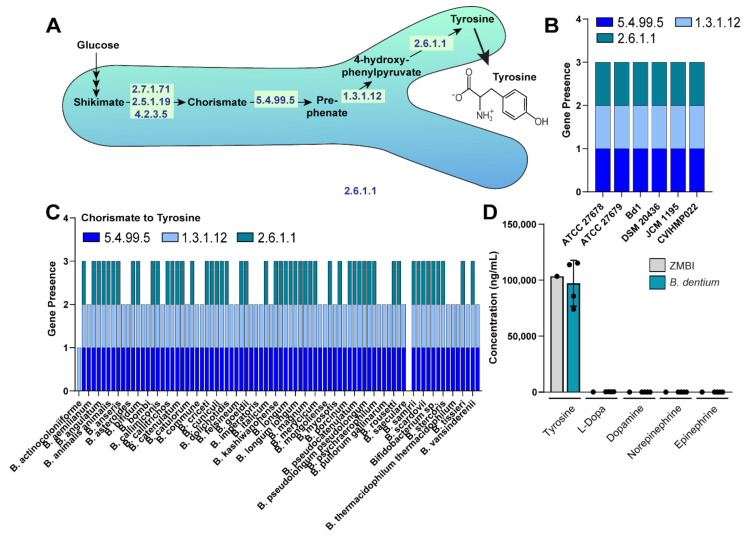
(**A**). Gene pathways in *B. dentium* as identified via KEGG (https://www.genome.jp/kegg/kegg2.html) related to tyrosine production. (**B**). Genome analysis of the JGI Integrated Microbial Genomes (IMG) database (http://img.jgi.doe.gov (accessed on 17 January 2021)) of six *B. dentium* genomes for the enzymes (Enzyme Commission, ECs) involved in tyrosine production. Data represent the number of copies for each EC gene. (**C**). Genome analysis of 83 *Bifidobacteria* genomes using the JGI Integrated Microbial Genomes (IMG) database. (**D**). Absolute concentrations of tyrosine, L-dopa, dopamine, norepinephrine, and epinephrine in uninoculated ZMB1 and cell-free bacterial conditioned ZMB1 from *B. dentium* after 18 h growth. *n*= 4 biological replicates, two-way ANOVA.

Next, we examined neurotransmitters in the dopamine pathway, including tyrosine, L-dopa, dopamine, norepinephrine, and epinephrine. These neurotransmitters have been shown to act on the enterochromaffin cells and modulate the ENS. We queried the *B. dentium* Bd1 genome using the KEGG database for the ECs associated with the production of tyrosine, L-dopa, dopamine, norepinephrine, and epinephrine. Analysis of the KEGG pathway revealed that *B. dentium* could convert chorismate into tyrosine through a three-step process (Figure 3A). However, no genes were identified which could generate L-dopa, dopamine, norepinephrine, or epinephrine. We next searched within the IMG database for the presence of these genes within several *B. dentium* genomes and found that all six *B. dentium* genomes, including the *B. dentium* ATCC 27678 strain used in our gnotobiotic studies, harbored the genes to convert chorismate into tyrosine (Figure 3B).

To determine if tyrosine pathway genes were conserved in the *Bifidobacteria* genomes, we examined the genomes of the 83 *Bifidobacteria* species available in the IMG database. Interestingly, 31 (49%) *Bifidobacteria* species did not have tyrosine aminotransferase (EC 2.6.1.1), which converts 4-hydroxy-phenylpyruvate into tyrosine, indicating that tyrosine production is species dependent. We did not observe any genes related to L-dopa, dopamine, norepinephrine, or epinephrine production in our genome analysis. Next, we examined tyrosine concentrations in our fully defined ZMB1 media (Figure 3C). ZMB1 at baseline contains high concentrations of tyrosine (103 µg/mL). Although we observed that *B. dentium* had the molecular machinery to produce tyrosine, we found that *B. dentium* did not generate tyrosine in ZMB1 using LC-MS/MS analysis. As expected, we found no production of L-dopa, dopamine, norepinephrine, and epinephrine, consistent with the absence of the required genes in the *B. dentium* genome.

### 3.4. Bifidobacterium Dentium ATCC 27678 Influences Fecal and Neuronal Tyrosine Levels in Gnotobiotic Animals

**Figure 4:**
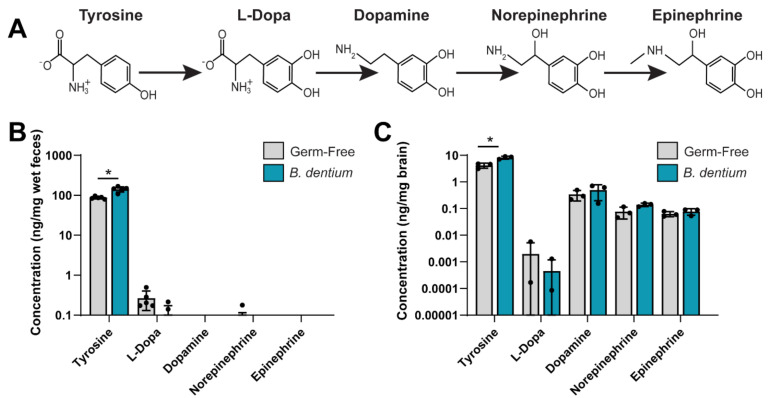
(**A**). Depiction of the tyrosine pathway. (**B**). Analysis of tyrosine, L-dopa, dopamine, norepinephrine, and epinephrine measured from a known mass of stool (ng/mg wet feces weight) by LC-MS/MS reflecting in vivo levels of these neurotransmitters from germ-free and *B. dentium* mono-associated mice. (**C**). Concentrations of tyrosine, L-dopa, dopamine, norepinephrine, and epinephrine in a known mass of whole brain homogenate (ng/mg brain). For all experiments, *n* = 5 mice/group; two-way ANOVA, * *p* < 0.05.

**Figure 5:**
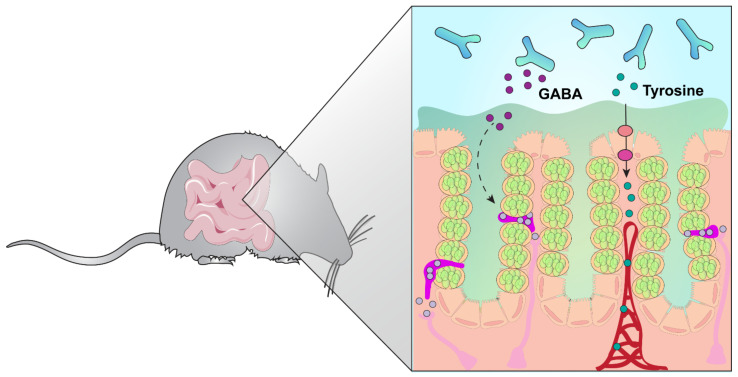
Model diagram of *B. dentium* colonization. Our data indicate that *B. dentium* can generate glutamine, GABA, and tyrosine. We speculate that GABA has a local effect on the enteroendocrine cells and enteric neurons in the gut, while tyrosine can be transported through the epithelium, enter the circulation, and be transported into the brain.

To address whether *B. dentium* could modulate tyrosine concentrations in vivo, we examined the tyrosine pathway in the stool of *B. dentium* mono-associated mice by LC-MS/MS and compared them with germ-free controls (Figure 4). We observed elevated levels of tyrosine in the feces of *B. dentium-*treated mice compared to the germ-free controls (Figure 4B). Tyrosine can be transported through epithelial cells and enter the circulation. Alternatively, tyrosine can enter cells such as enteric neurons and be converted to L-dopa and dopamine, and subsequently to norepinephrine and epinephrine (Figure 4A). Interestingly, no changes were observed in the other neurotransmitters in the stool samples. Since tyrosine can be transported through the blood–brain barrier, we examined the whole brain homogenate by LC-MS/MS (Figure 4C). Similar to our stool, we found that tyrosine was elevated in the brain of *B. dentium* mono-associated mice compared to the germ-free controls; without changes to the other neurotransmitters. Collectively, these data indicate that *B. dentium* colonization influences the levels of select gut neurotransmitters (Figure 5).

## 4. Discussion

Accumulating evidence indicates that the gut microbiota has a significant impact on the host physiology. One route by which microbes can influence the host is through the production or regulation of neurotransmitters. Herein, we characterize the production of important host-related neurotransmitters by the gut commensal *B. dentium*. In contrast to other microbes [59], we found that *B. dentium* was unable to synthesize or modulate dopamine, epinephrine, and norepinephrine. However, *B. dentium* was able to modulate intestinal concentrations of GABA and tyrosine in vivo. Additionally, we found that *B. dentium* colonization was associated with an increase in tyrosine in the whole brain homogenate. These findings demonstrate new pathways by which *B. dentium* can modify the host.

Gnotobiotic models serve as a simple exploratory technique to determine how individual microbes influence the host. Using this approach, we previously found that *B. dentium* metabolites stimulated the release of serotonin from intestinal enterochromaffin cells [52]. However, the complete profile of intestinal and neuronal neurotransmitters associated with *B. dentium* colonization was not examined. Herein, we attempted to address this knowledge gap by systemically characterizing stool and whole brain homogenates from germ-free and *B. dentium* mono-associated mice. We selected the stool as a simplified approach to examine microbial-produced or apically-released neurotransmitters. Because *B. dentium* was able to produce GABA in vitro in ZMB1 media, we believe that the increased levels of GABA in *B. dentium* mono-associated stool samples reflect microbially-produced GABA. According to our genome analysis, only 15% of *Bifidobacteria* harbor all the enzymatic machinery to generate GABA from all sources. We interpret this data to indicate that *B. dentium* is a more interesting *Bifidobacteria* candidate for the manipulation of GABA. In vitro, we also observed that *B. dentium* was able to secrete glutamine, which can enter intestinal epithelial cells via the ASCT2 transporters. Glutamine can be converted to glutamate and subsequently to GABA. Therefore, it is possible that host-derived GABA could contribute to the elevated fecal GABA levels. In the future, it would be interesting to examine how quickly *B. dentium* produces GABA in vitro and in vivo. This information may inform therapeutic approaches. Our KEGG and IMG pathways analysis revealed that *B. dentium* could generate glutamate and glutamine, but we did not see any changes in these compounds in our stool samples. Glutamine and glutamate are abundant in the diet, particularly in the diet of germ-free mouse chow, and we speculate that the high availability of these compounds in the intestinal lumen limited the production of these compounds by *B. dentium*. Additionally, it is possible that *B. dentium* may be consuming dietary glutamate and glutamine at the same rate as secretion; resulting in no net change in these amino acids. Further studies using stable-isotope labeled substrates would help shed light on the direct contribution of *B. dentium* to glutamine and glutamate production in vivo. Interestingly, although GABA, glutamate, and glutamine were found in high concentrations in the brain (~1000 ng/mg brain tissue), these levels were unchanged in response to *B. dentium* colonization. These data suggest that *B. dentium* colonization in the gut does not influence neuronal GABA concentrations.

Another interesting finding was that *B. dentium* elevated tyrosine concentrations in the gut and brain. Tyrosine is a non-essential amino acid that can be transported from the intestine into the circulation, cross the blood–brain barrier, and enter neurons, where it is metabolized into catecholamine neurotransmitters. We found that *B. dentium* harbored the genes to generate tyrosine. We did not observe elevated levels of tyrosine in our ZMB1, which had incredibly high tyrosine levels at baseline. We speculate that the high levels of tyrosine in the ZMB1 limited the production of tyrosine by *B. dentium* in vitro. Future work optimizing the concentration of tyrosine for growth and secretion would provide an interesting baseline for confirming bacterial tyrosine production. Although we did not observe tyrosine production in vitro, we observed elevated concentrations of tyrosine in the feces of *B. dentium*-associated mice when compared to the germ-free controls. Since epithelial cells do not appear to have a mechanism to apically secrete tyrosine, we believe that the elevated tyrosine levels in *B. dentium*-associated mouse stool samples reflect *B. dentium* synthesis of this amino acid. Although select facultative anaerobes such as *Bacillus*, *Escherichia*, and *Proteus* have been found to produce dopamine and norepinephrine [59], we did not observe detectable levels of dopamine, epinephrine, or norepinephrine in *B. dentium*-conditioned ZMB1 or in our *B. dentium* mono-associated mouse feces. These findings are consistent with our KEGG pathway analysis and indicate that *B. dentium* is unable to synthesize these neurotransmitters.

In vivo, tyrosine is an important regulator of the neuronal catecholamine neurotransmitters, including dopamine, epinephrine, and norepinephrine. Although we saw elevated tyrosine, we did not observe increased concentrations of these catecholamine neurotransmitters in the stool. We speculate that this is because dopamine-producing cells are not commonly found in the intestinal epithelium. Consistent with this notion, we have previously examined a panel of neurotransmitters in human jejunum organoids, also known as enteroids, transduced to increase enteroendocrine cells and we did not detect dopamine, epinephrine, or norepinephrine neurotransmitters [61]. Previous studies have shown that dopamine is largely synthesized in the brain [62]. We found moderate dopamine levels (~1 ng/mg brain tissue) in our germ-free and gnotobiotic mouse brain samples. Epinephrine and norepinephrine are largely produced in sympathetic nerve fibers or adrenal medulla [62]. We also observed moderate concentrations of epinephrine and norepinephrine in the whole brain homogenate. Although we observed increased tyrosine concentration in the *B. dentium* mono-associated mouse brains, we surprisingly did not see any changes in dopamine, epinephrine, or norepinephrine. This observation could be due to the fact that we examined whole brain neurotransmitter levels. Another possibility is that dopamine concentrations could be elevated in specific brain compartments, but these distinct levels are averaged in a whole brain homogenate. It is also possible that the neuronal cells which generate dopamine require additional metabolite signals generated by other gut microbes to convert tyrosine to dopamine. Finally, since our mice were colonized as adults, it may be that early-life colonization is necessary for the proper wiring of these circuits. Additional studies are necessary to fully tease out this information.

In addition to neurotransmitter production, there are several methods by which the gut microbiota can influence the gut–brain axis, including vagal nerve stimulation, activation of neuropods/direct stimulation of afferent nerves, altering the activity of the stress-associated hypothalamic–pituitary–adrenal (HPA) axis, regulation of the immune cells, and modulating the permeability of the blood–brain barrier [8,24,63,64,65,66,67,68,69,70,71,72,73,74]. Although we have not examined these pathways in our gnotobiotic mouse model, we believe that these routes may be important contributors to the *Bifidobacteria*–host modulation and should be explored in the future.

In conclusion, this work has provided a unique glimpse into neurotransmitter profiles using germ-free and mono-associated gnotobiotic animals. Our data is among the first to demonstrate that a single microbe is sufficient to modulate select gut and neuronal neurotransmitters in vivo. Previous work with gut microbes has revealed that many microbial effects are strain-specific. As a result, it is important to delineate which microbes are associated with certain effects. We speculate that mono-associated animals and LC-MS/MS analyses may allow researchers to dissect the essential pathways by which commensal microbes influence the gut–brain axis. A better understanding of these pathways may provide novel adjuvant strategies for both gastroenterological and neurologic disorders.

## Data Availability

Data available on request.

## Supplementary Materials

The following are available online at https://www.mdpi.com/article/10.3390/biom11081091/s1, Table S1: *Bifidobacteria* species identified in the JGI Integrated Microbial Genomes (IMG) database (http://img.jgi.doe.gov) (accessed on 17 January 2021). Table S2: SRM transition parameters for the glutamate cycle metabolites on the Sciex 6500 QTrap MS. Table S3: SRM transition parameters for the Tyrosine Pathway metabolites on the Sciex 6500 QTrap MS.
