# Supplementary material for: Neurotransmitter Profiles Are Altered in the Gut and Brain of Mice Mono-Associated with Bifidobacterium dentium
Source: Biomolecules. 2021 Jul 23;11(8):1091. doi: 10.3390/biom11081091 (PMC8392031; doi:10.3390/biom11081091)
Supplement: Supplementary file 1 [file biomolecules-11-01091-s001.zip › biomolecules-1265468-supplementary.pdf]

Supplemental Information: Neurotransmitters profiles are altered in the gut and brain of mice mono-associated with *Bifidobacterium dentium*

Authors: Berkley Luck[1,2*], Thomas D. Horvath[1,2*], Kristen A. Engevik[6], Wenly Ruan[3,4], Sigmund J. Haidacher[1,2], Kathleen M. Hoch[1,2], Numan Oezguen[1,2], Jennifer K. Spinler[1,2], Anthony M. Haag[1,2], James Versalovic[1,2], Melinda A. Engevik[1,7#]

Supplemental Methods:

Table S1: *Bifidobacteria* species identified in the JGI Integrated Microbial Genomes (IMG) database (http://img.jgi.doe.gov) (accessed 17Jan21).

Bifidobacteria species	Available Genomes
B. actinocoloniiforme	2
B. adolescentis	13
B. aemilianum	1
B. aesculapii	1
B. angulatum	4
B. animalis	3
B. animalis animalis	10
B. animalis lactis	24
B. anseris	1
B. aquikefiri	1
B. asteroides	10
B. biavatii	2
B. bifidum	33
B. bohemicum	3
B. bombi	2
B. boum	3
B. breve	68
B. callitrichos	1
B. callitrichidarum	1
B. callitrichos	3
B. castoris	1

B. catenulatum	4
B. catenulatum kashiwanohense	3
B. catulorum	1
B. choerinum	4
B. commune	2
B. coryneforme	4
B. criceti	1
B. crudilactis	1
B. cuniculi	2
B. dentium	7
B. dolichotidis	1
B. eulemuris	1
B. felsineum	1
B. gallicum	3
B. goeldii	1
B. hapali	1
B. imperatoris	1
B. indicum	2
B. italicum	1
B. jacchi	1
B. kashiwanohense	1
B. lemurum	2
B. longum	19
B. longum infantis	36
B. longum longum	100
B. longum suis	7
B. magnum	3
B. margollesii	1
B. merycicum	4

B. minimum	3
B. mongoliense	2
B. moukalabense	3
B. myosotis	2
B. parmae	1
B. porcinum	2
B. primatium	1
B. pseudocatenulatum	19
B. pseudolongum globosum	4
B. pseudolongum pseudolongum	2
B. pseudolongum	2
B. psychraerophilum	2
B. pullorum	2
B. pullorum gallinarum	3
B. reuteri	2
B. rousetti	1
B. ruminantium	3
B. saeculare	2
B. saguini	2
B. samirii	1
B. scaligerum	1
B. scardovii	4
B. simiarum	1
Bifidobacterium sp.	10
B. stellenboschense	1
B. stercoris	3
B. subtile	3
B. thermacidophilum thermacidophilum	3
B. thermophilum	4

B. tissieri	1
B. tsurumiense	3
B. vansinderenii	1
B. vansinderenii	1
B. xylocopae	1

LC-MS/MS Analysis

Chemicals, Reagents, and Durable Supplies

Optima™ LC/MS-grade water, acetonitrile (ACN), methanol, and acetic acid and formic acid (FA) were obtained from Fisher Scientific (Waltham, MA, USA). MS-grade ammonium acetate and ammonium formate, and ion-chromatography-grade heptafluorobutyric acid (HFBA) was from Millipore-Sigma (Burlington, MA, USA).

Authentic analytical references standards for metabolites measured in the targeted metabolomics method for the glutamate cycle include L-glutamine, L-glutamate, and GABA, and their authentic deuterated Internal Standard (IS) reference compounds including d5-L-glutamate and d6-GABA were all purchased from Millipore-Sigma, and d5-L-glutamine was purchased from CDN Isotopes (Pointe-Claire, Quebec, Canada). Chromatographic separations were performed using a Supelco Ascentis® Express HILIC (150 mm x 2.1 mm, 2.7 µm, 90 Å pore) analytical column from Millipore-Sigma.

Authentic analytical reference standards for metabolites in the Tyrosine Pathway include dopamine, epinephrine, levodopa (L-DOPA), D,L-norepinephrine, and L-tyrosine (Tyr) were all purchased from Millipore-Sigma. Deuterated IS reference compounds for d4-dopamine, d6-epinephrine, and d3-L-DOPA were all purchased from Millipore-Sigma. Chromatographic separations were performed using a Raptor C18 (100 mm x 1 mm, 2.7 µm, 90 Å pore size) analytical column equipped with an Ultra C18 (10 mm x 2.1 mm, 5 µm, 100 Å pore size) guard cartridge from Restek.

Homogenization and Metabolite Extraction Procedures for Fecal and Brain Samples

For neurotransmitter analysis of the brain, whole brain was homogenized and ~100 mg tissue was transferred to pre-weighed 2-mL Fastprep tubes containing 100 mg of Lysing Matrix D beads (1.4 mm ceramic spheres) and homogenized with ice-cold methanol:water (1:1, *v:v*; 0.1 mg/µL tissue density) on a FastPrep-24 Classic Bench-Top Bead Beating Lysis System. Brain tissue homogenates were centrifuged at 16,000 x *g* for 5 min, and the supernatant was transferred to new tubes and stored frozen at -80°C for downstream processing. For fecal analysis, an ~50 mg mass of fecal sample was weighed, mixed at a tissue density of 0.1 mg/µL in ice-cold methanol:water (1:1, *v:v*), homogenized by vortex-mixing for 5 min, centrifuged at 16,000 *x g* for 5 min, and the supernatant was transferred to new tubes and was stored frozen at -80˚C for downstream processing.

LC-MS/MS Method for Glutamate Cycle Metabolites

Critical Solution Preparations:

Internal Standard (IS) stock solutions for d5-glutamate, d5-glutamine, and d6-GABA were each prepared at 10.0 mg/mL in water. An IS Solution-A (ISS-A) was prepared from each of the IS stocks at a concentration of 500 ng/mL for each in a solution of acetonitrile:water (9:1, *v:v*) and 0.1% formic acid; the ISS-A solution was used in the final preparation of the microbiome samples. An ISS-B solution was prepared at a concentration of 450 ng/mL for each deuterated internal standard compound by diluting a 9-mL volume of the ISS-A solution with a 1-mL volume of acetonitrile:water (9:1, *v:v*) and 0.1% formic acid;

This solution was used to prepare the combined intermediate solution and the Calibration Standards (Calibrators).

Stock solutions for glutamate, glutamine, and GABA were each prepared at 10.0 mg/mL in water. A combined intermediate was prepared from each of the stocks at concentrations of 100 µg/mL for each using Glutamate Cycle ISS-B as the diluent. Calibrators were prepared from the combined intermediate by serial dilution (dilution factor of 4-fold per Calibrator level) at metabolite concentrations of 1,000, 250, 62.5, 15.6, 3.90, and 0.977 ng/mL using Glutamate Cycle ISS-B as the diluent.

Microbiome Sample Preparations for the Glutamate Cycle Metabolites:

Homogenized stool and brain sample extracts were thawed on the benchtop at ambient room temperature, and were vortex-mixed briefly. A 10-µL volume of stool or brain homogenate sample extract was diluted in a 90-µL volume of the Glutamate Cycle ISS-A solution directly in an autosampler vial, and the 10-fold diluted samples were vortex-mixed for 30 sec. The samples were transferred to an autosampler and a 5-µL sample volume was injected onto the LC-MS/MS system for analysis.

LC-MS/MS Conditions and Parameters for the Glutamate Cycle Targeted Method:

The Glutamate Cycle method uses hydrophilic interaction chromatography (HILIC) separation for the measurement of the glutamate, glutamine, and GABA. Chemical separations were performed using a mobile phase A (MPA) solution consisting of ACN:water (9:1, *v:v*) and 10 mM ammonium formate and 2% FA, a mobile phase B (MPB) solution consisting of ACN:water (1:1, *v:v*) and 10 mM ammonium formate and 2% FA, and a needlewash solution (NW) consisting of ACN:water (1:1, *v:v*). The mobile phase flowrate was 0.200 mL/min, the autosampler trays were chilled to 4°C, the column oven was heated at 30°C, and the gradient elution program used was 0-2.5 min, 0% MPB; 2.5-10.5 min, 0-50% MPB; 10.5-13.0 min, 50% MPB; 13.0-14.0 min, 50-0% MPB; 14.0-17.0 min, 0% MPB with a gradient cycle time of 17.4 min per sample. A TurboIonSpray® electrospray ionization (ESI) probe was installed and the 6500 QTrap MS was operated in positive ionization mode using a selected-reaction monitoring (SRM) scan mode under the following instrumental conditions: IonSpray voltage of +5,000 volts (V); Curtain gas (Cur): 20 psi; Temperature (Temp): 200°C; Source Gas 1 (GS1): 25 psi; Source Gas 2 (GS2): 25 psi; Collisionally-activated dissociation (CAD) gas: HIGH; and the Q1 and Q3 quadruple resolution settings were set to Unit/Unit. See Table S1 for the metabolite specific SRM transition parameters for the glutamate cycle metabolites.

Table S1: SRM transition parameters for the glutamate cycle metabolites on the Sciex 6500 QTrap MS:

Metabolite	Q1 (*m/z*)	Q3 (*m/z*)†	DP (V)[a]	EP (V)[b]	CE (eV)[c]	CXP (V)[d]
GABA	104.1	87.1 / 69.1	80	9	20 / 30	9
d_6-GABA (IS)	110.1	92.1 / 73.1	80	9	20 / 30	9
glutamate	148.1	84.0 / 130.1	55	9	24 / 14	11
d_5-glutamate (IS)	153.1	88.1 / 135.1	55	9	24 / 14	11
glutamine	147.1	130.1 / 84.1	55	9	14 / 24	11
d_5-glutamine (IS)	152.1	135.1 / 88.1	55	9	14 / 24	11

†The black text corresponds to the mass-to-charge (*m/z*) of the quantifying fragment ion, and the red text corresponds to the *m/z* of the qualifying fragment ion. Abbreviations: [a]DP, declustering potential; [b]EP, entrance potential; [c]CE, collision energy; [d]CXP, collision-cell exit potential; V, volts; eV, electron volts.

LC-MS/MS Method for the Quantitation of Tyrosine Pathway Metabolites
Critical Solution Preparations:

IS stock solutions for d4-dopamine, d6-epinephrine, and d3-levodopa (L-DOPA) were each prepared at 10.0 mg/mL in water. An ISS-A solution was prepared from each of the IS stocks at a concentration of 125 ng/mL for d4-dopamine, 200 ng/mL for d6-epinephrine, and 1,000 ng/mL for d3-L-DOPA in water; the ISS-A solution was used in the final preparation of the microbiome samples. An ISS-B solution was prepared at a concentration of 112.5 ng/mL for d4-dopamine, 180 ng/mL for d6-epinephrine, and 900 ng/mL for d3-L-DOPA by diluting a 9-mL volume of the ISS-A solution with a 1-mL volume of water; this solution was used to prepare the combined intermediate solution and the Calibrators.

Stock solutions for dopamine, epinephrine, L-DOPA, norepinephrine, and tyrosine were each prepared at 10.0 mg/mL in water. A combined intermediate was prepared from each of the stocks at concentrations of 100 µg/mL for each using Tyrosine Pathway ISS-B as the diluent. Calibrators were prepared from the combined intermediate by serial dilution (DF 4-fold per Calibrator level) at metabolite concentrations of 1,000, 250, 62.5, 15.6, 3.90, and 0.977 ng/mL using Tyrosine Pathway ISS-B as the diluent.

Microbiome Sample Preparations for the Tyrosine Pathway Metabolites:

Homogenized stool and brain sample extracts were thawed on the benchtop at ambient room temperature, and were vortex-mixed briefly. A 10-µL volume of stool or brain homogenate sample extract was diluted in a 90-µL volume of the Tyrosine Pathway ISS-A solution directly in an autosampler vial, and the 10-fold diluted samples were vortex-mixed for 30 sec. The samples were transferred to an autosampler and a 5-µL sample volume was injected onto the LC-MS/MS system for analysis.

LC-MS/MS Conditions and Parameters for the Tyrosine Pathway Targeted Method:

The ion pairing-based reverse-phase separation for the measurement of Tyrosine Pathway metabolites was performed using a MPA solution consisting of water:ACN:FA:HFBA (99.3:0.5:0.1:0.1, *v:v:v:v*), a MPB solvent consisting of 100% ACN, and a NW consisting of ACN:water (1:1, *v:v*). The mobile phase flowrate was 0.200 mL/min, the autosampler trays were chilled to 10°C, the column oven was heated at 40°C, and the gradient elution program used was 0-0.5 min, 5% MPB; 0.5-6.0 min, 5-90% MPB; 6.0-7.0 min, 90% MPB; 7.0-7.1 min, 90-5% MPB; 7.1-12.0 min, 5% MPB with a gradient cycle time of 12.4 min per sample. An ESI probe was installed and the 6500 QTrap MS was operated in positive ionization mode using an SRM scan mode with the following instrumental conditions: IonSpray voltage of +5,000 V; Cur: 20 psi; Temp: 300°C; GS1/GS2: 25 psi each; CAD gas: HIGH; and, Q1/Q3 resolution: Unit/Unit. See Table S2 for the metabolite specific SRM transition parameters for the Tyrosine Pathway metabolites.

Table S2: SRM transition parameters for the Tyrosine Pathway metabolites on the Sciex 6500 QTrap MS:

Metabolite	Q1 (*m/z*)	Q3 (*m/z*)[†]	DP (V)[a]	EP (V)[b]	CE (eV)[c]	CXP (V)[d]
tyrosine	182.1	165.1 / 136.1	50	8	14 / 19	10
dopamine	154.1	137.1 / 91.1	40	9	15 / 32	10
d_4-dopamine (IS)	158.1	141.1 / 95.1	40	9	15 / 32	10
L-DOPA	198.1	181.1 / 152.1	50	7	14 / 20	9
d_3-L-DOPA (IS)	201.1	184.1 / 155.1	50	7	14 / 20	9
norepinephrine	170.1	152.1 / 107.1	40	9	12 / 26	9
epinephrine	184.1	166.1 / 151.1	40	8	15 / 30	15
d_6-epinephrine (IS)	190.1	172.1 / 157.1	40	8	15 / 30	15

[†]The black text corresponds to the mass-to-charge (*m/z*) of the quantifying fragment ion, and the red text corresponds to the *m/z* of the qualifying fragment ion. Abbreviations: [a]DP, declustering potential; [b]EP, entrance potential; [c]CE, collision energy; [d]CXP, collision-cell exit potential; V, volts; eV, electron volts.
